# Case of Fracture of the Occipital Bone, with Lesion of the Brain, Successfully Treated

**Published:** 1830

**Authors:** John Ament Haldeman

**Affiliations:** Fayette, Missouri


ART.—II. *Case of fracture of the Occipital Bone, with Lesion of the Brain, successfully treated:* By JOHN AMENT HALDEMAN, M. D., of Fayette, Missouri.

B. F. Mead, a girl of sprightly disposition, in the 4th year of her age, was kicked on the back part of the head by a horse, on the 15th of April last. As the patient lived five miles in the country, it was nearly three hours after the reception of the injury, before I saw her. On my arrival, I found her covered with blood from the wound, in a state of complete insensibility; respiration slow and laborious; eyes vacant, with dilated pupils; pulse slow, regular, and rather full; voluntary motion completely suspended, without convulsion or spasm; skin florid, moist, and several degrees below the healthy temperature.

On examining the wound, I found the occipital bone extensively fractured and depressed, with an area corresponding to the horses hoof, the lower curvature of the depression being just above the occipital cross, the wound bleeding freely, and the comminuted brain discharging through the crevices of the bone.

From a sense of professional duty, rather than from any hope of success, I determined to remove the portions of bone that were pressing on the cerebrum. Accordingly, having washed away the coagulated blood, and cut off the hair as closely as possible with a pair of scissors, I made an incision two inches long from the middle of the wound to the centre of the semicircular depression, dissected up the flaps, and removed sixteen spiculæ of bone. On the removal of the bone, the little patient began to manifest some signs of sensibility, indicated by a slight inclination, accompanied with an indistinct muttering call to her mother, who now took and held her during the remainder of the operation. The surface of the dura mater exposed, was about equal to a French crown, extending from the transverse sinus upwards to the junction of the occipital and parietal bones.—The dura mater was entire, except at the lower part of the wound, where it was lacerated to an extent sufficient to admit of a luxuriant growth of "hernia cerebri," which, in the progress of the cure, proved very troublesome. Several large pieces of bone which adhered at the upper extremity, but below were much depressed, were elevated, and suffered to remain, and all healed favorably, although from one piece the external table was removed. The flaps were drawn over the whole of the dura mater which was uninjured, and retained by strips of adhesive plaster. The rest of the wound was dressed with pledgets of dry lint, and allowed to suppurate; the whole covered by a roller bandage.

The incision made for the purpose of removing the bone, healed by the first intention. Small quantities of brain were discharged, and a considerable flow of blood took place at several of the first dressings, and during the cicatrization, numerous spiculæ of bone exfoliated, and were thrown off. The discharge from the wound, at first dark and serous, assumed, in the course of six or eight days, the appearance of laudable pus, but about this time, fungus cere bri, began to appear, which, eventually, proved a source of great difficulty and embarrassment.

Two weeks after the injury, a portion of the inner table of the os occipitis, about as wide, and a little longer than a man's thumb, exfoliated, and was removed without injuring the membrane over the cerebellum. This fragment, which was removed from the centre of the occipital bone, must have extended half an inch below the occipital ridge, and the fact may seem to show the error of the opinion once entertained, that wounds of this part are necessarily mortal.

The general treatment pursued, was very simple. Active catharsis was kept up for a few days, by salts and senna, and a system of the most rigid abstinence was enjoined, and strictly enforced. No inflammatory symptoms supervened, nor any derangement of the digestive functions, and after the effects of the concussion and compression had subsided, the mental faculties were completely restored, and the patient manifested a degree of sprightliness and intelligence, very unusual in one of her age. The upper part of the wound healed by the first intention; and the lower part began to secrete healthy pus, and to put forth healthy granulations, about the sixth or eigth day. About this time, fungus cere bri began to appear, the difficulty of subduing which, although I was aware that the tendency to this morbid growth, in organic lesions of the brain, was equalled only by the professional attendance necessary to suppress it, far exceeded all my expectations. By the frequent application of pressure with the dry sponge, according to the directions of Professor Dudley,* I was enabled effectually to check, and reduce it. On two occasions, I had recourse to the knife, not so much with view to its entire removal, as to ascertain the relative merits of the two modes of practice. No unfavorable symptoms supervened; nothing that would indicate the impropriety of removing the entire tumour by the scalpel, but as the application of the sponge was less terrifying, and equally effectual, it was afterwards exclusively relied upon. In the course of her recovery, the wound became somewhat indolent, and occasionally required stimulating applications, an indication which was effectually fulfilled by the peroxide of mercury. The wound was perfectly healed in less than three months, without any other untoward symptom.

When we consider the violence, the seat, and the extent of this injury, we look upon the perfect recovery, with the uninterrupted healthful play of the mental faculties, and the entire absence of any unfavorable constitutional symptoms, as a very unusual occurrence; and as such, not unworthy of being recorded. In con-

---

* Transylvania Journal of Medicine, Vol. I. No. 1.

clusion we will take the liberty of remarking that the influence which this injury may have on the organ of philoprogenitivenesss may form an interesting object of inquiry to the phrenologist; and at some future day the development of these organs may convey some useful information, and call forth much ingenuous speculation upon this subject.

*Editorial Remarks.*—We publish the above case, as well from its interesting character, as from a wish to direct the attention of the younger members of the profession to the subject of injuries of the head generally. From the greater thickness and strength of the occiput, and the proportionate degree of violence requisite to fracture the skull in this part; the vicinity of the spinal marrow, and of the origin of those nerves which preside over the most important functions of the animal economy; and the dense covering of muscles and fasciæ with which this part is covered, injuries of the cerebellum are attended with much more danger than any of the other parts of the brain. The mildness of the symptoms in the present case, may, perhaps, in some degree be attributed to the fact, that from the imperfect ossification of the cranium, the fracture of the occiput, in this instance, was attended with less concussion of the brain, than must necessarily have accompanied so extensive an injury at a latter period of life.

To young physicians we would particularly recommend the study of the surgical anatomy of the head, and the acquisition of that practical skill which is necessary to enable them to meet those emergencies which are perpetually liable to occur to physicians in the country, in which they are compelled to operate without professional advice or assistance. As an additional incentive to the student, in the study of this particular branch of his profession, it may be urged that while the great importance of the brain calls for the most prompt and skilful treatment, in no other case are the requisite information and manual dexterity more completely within the reach of every practitioner, or are the rules of practice, notwithstanding the uncertainty of the result in particular cases, defined with more accuracy and precision.

